# Introduction to Semi-Classical Analysis for Digital Errors of Qubit in Quantum Processor

**DOI:** 10.3390/e23121577

**Published:** 2021-11-26

**Authors:** Osamu Hirota

**Affiliations:** 1 Quantum ICT Research Institute, Tamagawa University, Tokyo 194-8610, Japan; hirota@lab.tamagawa.ac.jp; 2 Reserch and Development Initiative, Chuo University, Tokyo 112-8551, Japan

**Keywords:** communication channel error model, nonlinear error, burst error, cosmic ray, quantum Zeno effect

## Abstract

In recent years, remarkable progress has been achieved in the development of quantum computers. For further development, it is important to clarify properties of errors by quantum noise and environment noise. However, when the system scale of quantum processors is expanded, it has been pointed out that a new type of quantum error, such as nonlinear error, appears. It is not clear how to handle such new effects in information theory. First of all, one should make the characteristics of the error probability of qubits clear as communication channel error models in information theory. The purpose of this paper is to survey the progress for modeling the quantum noise effects that information theorists are likely to face in the future, to cope with such nontrivial errors mentioned above. This paper explains a channel error model to represent strange properties of error probability due to new quantum noise. By this model, specific examples on the features of error probability caused by, for example, quantum recurrence effects, collective relaxation, and external force, are given. As a result, it is possible to understand the meaning of strange features of error probability that do not exist in classical information theory without going through complex physical phenomena.

## 1. Introduction

When an ideal architecture of quantum processor is available, quantum computers are theoretically predicted to have significantly higher computing power than conventional computers [1]. The Shor’s algorithm is a prime example of its appeal, because it will bring the jeopardization of public key cryptography. A few years ago, the standardization of quantum computer-resistant cryptography [2] began in conjunction with the development of the quantum computer. In general, a quantum computer must consist of a combinatorial circuit of large number of quantum processors such as quantum gates and quantum memories. The recent demonstration of quantum transcendence by Google and IBM under the small number of qubits have sparked public interest in the real performance of quantum computers. Many people therefore expect a new prediction law based on the so called threshold theorem [3], that is comparable to Moore’s law for classical computers. According to the theorem, the errors in quantum computers can be corrected by several quantum error correcting schemes [4,5,6,7,8], and it will take a similar evolutionary process to the classical computer from the current small-scale quantum computer. However, the situation is not so simple. Furthermore, one should clarify the characteristics of quantum noise from the viewpoint of information theory. In this article, I will demonstrate how to achieve this. I firstly present the most general classification of potential errors by quantum noise. From such a classification, one can see a new kind of error that the error probability of a qubit depends on number of qubits in a quantum processor. This comes from the quantum nature, and has been called “Nonlinear error” [9,10]. I then introduce concrete physical phenomena for such features discussed in physics articles [11,12,13,14]. However, these phenomena are very complicated, and therefore, it is worthwhile to build an error model as an error in the communication channel model that can be understood without the detailed physical phenomena. Based on discoveries of a new type of error in the physics, I give the channel error model of error performance due to new quantum noise effects, and show several examples. This means to clarify properties of error probability, and it does not mean to discuss the concrete error such as bit error, phase error, and so on. This is important because there such strange properties of error probability do not exist in classical information theory [15,16,17], and such properties are also not taken into account in quantum error correction theory [4,5,6,7,8].

Let us here give the contents in this paper. Section 2 gives a classification of errors due to quantum noise effects in the information theoretic view. Section 3 introduces the basic equations for quantum noise analysis. Section 4 gives a review on physical examples of new quantum errors described in Section 2, which have been discovered recently. Section 5 shows concrete examples of strange features of error probability based on communication channel modeling. This provides a visualization of the strange error phenomena to researchers in information theory. Section 6 gives the error model and its features when the external force comes from the outside of the quantum processors, such as cosmic rays. Section 7 discusses an effect of the Quantum Zeno operation by external forces.

## 2. Information Theoretic View of Quantum Error

In a real system, the quantum computer operates as a time evolution of quantum states with noise, which brings an error in the digital processing. Therefore, one needs a method to mathematically model digital error due to the physical evolution of quantum states with noise. In this section, I classify the quantum noise effect by physical phenomena and give the corresponding information theoretic representation in order to make it easier for information theorists to participate. Here the information theoretic view of quantum error means to classify properties of error probability.

### 2.1. Phenomenal Classification of Quantum Noise

The quantum noise we are discussing here refers to the phenomenon of decoherence to quantum states. I list the classification of quantum noise in physics literature [18]:

1. Stochastic Pauli Noise: This corresponds to bit or phase flip errors of a single qubit.
2. Coherent Noise: No decoherence to a quantum state occurs, but it becomes an unintended quantum state.
3. Amplitude Damping: A specific example of decoherence, especially derived from energy loss.
4. Local correlated noise (Markov, non-Markov): This is an extension of Pauli noise, in which several qubits around the errored qubits are correlated to produce the error.
5. Non-local correlated noise (Markov, non-Markov):This has a potential to give an error for every qubit in the system with correlation.
6. The disentanglement noise: When the entanglement is released, it can be regarded as an error. These can be observed in an interaction with the environment, an interaction with other qubits, an imperfect gate, and also leakage, respectively. The details of these physical phenomena have been analyzed in physics. A list of references and a brief description of them are given in Appendix A.

The categorization of such noise for the information theory is important in order to proceed with a system design of the quantum computer. In particular, the effect of correlation based not only quantum but also on classical phenomena in quantum computing may cause a new type of error in the information theoretic view. The necessity of considering the correlated noise in the case of quantum computers is due to the following reasons. In the classical system, the semiconductor elements that make up a bit can be considered to be independent of each other. Next, the noise is additive, and errors in the execution of logical calculations are sufficiently practical to be analyzed only by the stochastic properties of the noise itself. As a result, almost all errors can be considered to be each bit independent or, if correlated, very local. On the other hand, in a quantum computer, some qubits are combined by quantum correlations such as entanglements, so it may be unique that only some qubits make errors independently.

### 2.2. Information Theoretic Classification of Quantum Errors

In this subsection, I present an information theoretic classification of errors that occur in quantum computers, which is the main subject of this paper. In information theory, the probability of the occurrence of an error is an important parameter. The detailed nature of the physical phenomena that cause errors is not the main subject. Therefore, the errors due to quantum noise mentioned in the previous section can be modeled based on the characteristics of the error probability, depending on what properties the quantum error has.

#### 2.2.1. Linear Individual Independent Error

Assume that *N* qubits are prepared. Errors shall occur separately and independently of each qubit. The basic error probability is set as 0≤p(error)≤1/2 in the information theory. The worst case is p(error)=1/2 . When this probability does not depend on the number of qubits, it is referred to as linear. The single error probability and a basis of *T* error probability in *N* qubits are given as follows, respectively:

(1) $$\begin{array}{ccc} & & p\left(error\right)\equiv \eta_{j}=\eta^{*}\;\forall j\in N \end{array}$$

(2) $$\begin{array}{ccc} & & P_{e}\left(T\right)\propto {\eta^{*}}^{T}{\left(1-\eta^{*}\right)}^{N-T} \end{array}$$

This is a standard error model in conventional information theory.

#### 2.2.2. Nonlinear Individual Independent Error

Let us assume that *N* qubits are prepared and that errors occur separately and independently in each qubit. If the error probability of *j*th qubit depends on the number of qubits, it is referred to as the nonlinear error. It can be defined as follows:

(3) $$p\left(error\right)=f\left(\eta^{*},N\right)\equiv \eta_{j}\left(N\right)$$

where this means that the error probability is a function of a number of qubits *N* and an error rate $\eta^{*}$ when only one qubit is prepared. When the concrete physical phenomena are analyzed, the above may be described as an approximation in some numerical regions for a physical setting as follows:

(4) $$p\left(error\right)∼\eta^{*}N^{\alpha }<\frac{1}{2}$$

This is valid for $\eta^{*}\ll 1$ . α is a real number to approximate the representation of the feature of *N* dependence in the error probability. This nonlinear error is the most serious error in quantum computers. The physical examples will be introduced in Section 4.

#### 2.2.3. Simple Burst Error Due to Correlation Phenomena

##### 2.2.3.1. Linear Local Correlated and Non-Local Correlated Error

Assume that *N* qubits are prepared, and the subset *T* qubits are correlated to each other. Let us assume that one qubit of *T* qubits decays with the probability $\eta^{*}$ , and let us assume that it does not depend on the number of qubits. However, if the *T* qubits collapse simultaneously due to one qubit decay, it is defined as a simple burst error. It is induced by correlation of neighboring (local) or arbitrary (non-local) qubits of the system. Then, the probability for the simple burst error is simply equal to the probability of one qubit error $\eta^{*}$ , as follows:

(5) $$P\left(burst\right)=\eta^{*}$$

This is the most simple description for burst error.

##### 2.2.3.2. Nonlinear Local or Non-Local Correlated Error

*N* qubits are prepared. Let us assume that an error occurs in one qubit, ands then the error occurs in the neighborhood or the whole system by correlation. In addition, the probability of the error of a single qubit triggering it depends on the number of qubits, as in Equation (3), and the nonlinear burst probability can be described by

(6) $$P\left(burst\right)=\eta_{j}\left(N\right)=f\left(\eta^{*},N\right)$$

This is called the nonlinear correlated error.

#### 2.2.4. Avalanche Burst Error and Accumulation Error

If one qubit makes an error, and the surrounding qubits make errors one after another based on classical correlation, I name this phenomenon an avalanche burst error. Such an error will be generated in a superconducting quantum computer when cosmic rays are irradiated to the system. Physical examples are discussed in Section 6. When the first error propagates to the next step in an iterative gate operation, or in an iterative calculation, with the assumption that the error accumulates in the quantum circuit, I define these phenomena as a propagation-accumulation error.

## 3. Basis of Quantum Noise Analysis

A quantum computation mechanism has a structure in which qubits in a QPU quantum processor unit are correlated, and a huge pure quantum state consisting of all qubits is unitarily evolved according to a program using the correlation. In other words, the whole QPU is considered to be monolithic. Therefore, the interaction between the pure state system and the environment, including the vacuum field, will inevitably cause the quantum states that carry information to become undesired quantum states, or to be destroyed. Then, simple bit-flip and phase flip (Pauli-flip type) errors similar to classical systems are rather exceptional, and quantum-specific errors can be the main ones. Therefore, in order to predict the realization of a large scale quantum computing mechanism, it is essential to elucidate the exact features of the noise itself by quantum noise analysis. The following is a starting point for this.

First, let *X* be a physical quantity representing a quantum bit, and let $N_{o}$ be the noise operator representing the noise to a quantum bit. Here the interaction between qubits and the interaction between qubits and noise are quite different from the classical system. The analysis of characteristics of these interactions is called quantum noise analysis. The interaction is denoted by the interaction Hamiltonian $H_{int}$ , which consists of *X* and N . The Hamiltonian of the entire system is

(7) $$H=H_{X}+H_{N}+H_{int}\equiv H_{0}+H_{int}$$

The quantum state representing the information evolves in time driven by the above Hamiltonian, but depending on the situation, either the Schr $\ddot{o}$ dinger equation on the extended Hilbert space or the following Lindblad equation [19]

(8) $$\frac{\partial \rho }{\partial t}=\frac{-i}{\hbar }\left[H,\rho \right]+\sum_{n=1}^{N^{2}-1}\left(v_{n}\rho v_{n}^{†}-\frac{1}{2}\left\{v_{n}^{†}v_{n},\rho \right\}\right)$$

is employed, where $v_{n}$ is a Lindblad-decoherence operator. Currently, this equation is the most frequently utilized.

Assuming an actual general purpose program, further generalization to include measurement systems is needed, not only models of decoherence systems as described in the previous section. As a generalization, a generalized stochastic Schr $\ddot{o}$ dinger equation [20] may be applicable. However, it is very hard to handle for calculation. Therefore, I simply employ the semi-classical stochastic differential equation, which is a simplification of the generalized stochastic Schr $\ddot{o}$ dinger equation. See Appendix A.

## 4. Review of Physical Examples of a New Type of Quantum Noise

This section introduces physical examples [11,12,13] of the error model categorized in Section 2. However, since physical phenomena are so complex, we exclude physical rigor and emphasize the logic that arrives at each error model. That is, the main objective is to show that the strange error characteristics in Section II exist in reality, or rather, that they may be the main noise in a certain type of quantum computers.

### 4.1. Hutter-Loss Recurrence Effect

Let us consider a model in which a group of qubits combined by quantum correlations, such as surface code, are interacted to the heat bath of a considered environment. There are many physical mechanisms available, such as direct interactions between each qubit and the heat bath, or non-Markovian interactions mediated by the heat bath. However, let us focus on the simplest of phenomena. That is, only bit-flip $\left(\sigma^{x}\right)$ for *X* is subject to error, the heat bath is in thermal equilibrium at the onset with the Hamiltonian $H_{N}=\sum_{k}\hbar \omega_{k}a_{k}^{†}a_{k}$ . Then the interaction Hamiltonian may be given by

(9) $$H_{int}=\sum_{j}\sigma_{j}^{x}⊗\sum_{\left\{k\in k\right\}}{\left|k\right|}^{r}\frac{\lambda }{\sqrt{M}}\left(e^{ikR_{j}}{\hat{a}}_{k}+e^{-ikR_{j}}{\hat{a}}_{k}^{†}\right)$$

$R_{j}$ means the spatial position of the qubit, *M* is the total number of modes, k∈k is the wave number of modes, r=0,±1/2 . Next, consider how a given *j*th qubit evolves as it interacts with the heat bath, where *j* of $R_{j}$ is the number of modes. If the initial state of the system is $\rho_{S}⊗\rho_{N}$ , then its decoherence evolution is expressed by using the Lindblad equation Equation (8) as follows [13]:

(10) $$\rho_{S}⟼\Phi_{e}\left(\rho_{S}\right)=Tr_{N}\left\{e^{-iHt}\left(\rho_{S}⊗\rho_{N}\right)e^{iHt}\right\}$$

$\rho_{S}$ is a density operator of all signal systems connected by correlation. Here, the operation in the measurements is added, such as

(11) $$\Psi_{\Pi }\left(\sigma \right)=\sum_{a}\Pi_{a}\sigma \Pi_{a}$$

Then the density operator for *j*th qubit becomes as follows:

(12) $$\rho_{j}\left(t\right)=Tr_{k\neq j}\circ \Psi_{\Pi }\circ \Psi_{e}\left(\rho_{S}\right)=\left(1-\eta_{j}\left(t,N\right)\right)\rho_{j}+\eta_{j}\left(t,N\right)\sigma_{j}^{x}\rho_{j}\sigma_{j}^{x}$$

where $\eta_{j}\left(t,N\right)$ is the error probability of the *j*th single qubit in a population of *N* qubits. From this formula, for a set with quantum correlations, the influence from all other qubits result in the following properties.

(13) $$\eta_{j}\left(t,N+1\right)=\cos^{2}\left(J_{1,N+1}\right)\eta_{j}\left(t,N\right)+\sin^{2}\left(J_{1,N+1}\right)\left(1-\eta_{j}\left(t,N\right)\right)$$

where

(14) $$J_{m,n}∼\lambda^{2}\int dk\frac{{\left|k\right|}^{2r}}{\omega_{k}^{2}}\times \cos\left(k\left(R_{m}-R_{n}\right)\right)\left(\sin\left(\omega_{k}t\right)-\omega_{k}t\right)$$

As a result, Hutter-Loss pointed out [13] that the error probability for *j*th qubit can be described by

(15) $$p\left(error\right)\propto \eta_{j}\left(t,N\right)\equiv f\left(t,\eta^{*},N\right)$$

The specific form of the dependency of *N* in the above equation is given by the formulae in Equation (34) based on the semiclassical analysis. Thus, this phenomenon induces a *N*-dependence of the error probability. Here I refer it as Hutter–Loss effect.

### 4.2. Collective Decoherence Effect

Here I would like to introduce another example for a new type of quantum noise. In general, one can consider a collective decoherence such as generalized Dicke super radiation. Let *N* atoms of a two-level system be qubits. Then one can assume more general discussion than the standard assumption that the wavelength of the radiation field is longer than the size of the qubit population in the interaction with the continuous mode field. The system can therefore be in the super radiant region. In general, the Wigner–Weisskopf theory is applied [21], and the interacting Hamiltonian, such as generalized Dicke super radiation, is given as follows:

(16) $$H_{int}=-\sum_{j}\sum_{n}\hbar \kappa_{n}\left({\hat{a}}_{n}\sigma {\left(+\right)}_{j}+{\hat{a}}_{n}^{†}\sigma {\left(-\right)}_{j}\right)$$

where $\sigma {\left(z\right)}_{j}={|e\rangle }_{jj}\langle e|-{|g\rangle }_{jj}\langle g|$ , $\sigma {\left(+\right)}_{j}={|e\rangle }_{jj}\langle g|$ , $\sigma {\left(-\right)}_{j}={|g\rangle }_{jj}\langle e|$ . Initially, the qubit system is assumed to be superimposed and the field is a vacuum. The initial state of the two coupled systems is given by

(17) $$|\Psi \left(t=0\right)\rangle =\sum_{m=0}^{2^{N}-1}c_{m,0}|m,0\rangle$$

Here let us show a physical analysis given by Lemberger and Yavus [11,12]. From the Schr $\ddot{o}$ dinger equation in the extended Hilbert space of the coupled system, the time evolution is

(18) $$|\Psi \left(t\right)\rangle =\sum_{m=0}^{2^{N}-1}c_{m,0}\left(t\right)e^{-i(N_{m}\omega_{a})t}|m,0\rangle +\sum_{n}\sum_{m^{\prime }}^{2^{N}-1}c_{m^{\prime },n}\left(t\right)e^{-i(N_{m^{\prime }}\omega_{a}+\nu_{n})t}|m^{\prime },1_{n}\rangle$$

It can be assumed that $N/2+\bar{N}$ are the excited state and $N/2-\bar{N}$ are the ground state among *N* qubits. $\bar{N}$ is the average number. The equation of motion for the stochastic amplitude of the point of interest in the above equation is given as follows.

(19) $$\frac{dc_{m,0}}{dt}=-\left(\frac{\Gamma }{2}+\delta \omega \right)\left(\frac{N}{2}+\bar{N}\right)\left(\frac{N}{2}-\bar{N}+1\right)c_{m,0}$$

where Γ and δω are the single decay rate and Lam shift, respectively. From the above, the decay of the probability amplitude of the representative point of interest is as follows.

(20) $$|c_{m,0}{\left(t\right)|}^{2}∼{\left|c_{m,0}\left(t=0\right)\right|}^{2}e^{-(N^{2}/4)\Gamma t}$$

The above equation is applicable to the majority of stochastic amplitudes for *N* qubits and it represents a feature of super-radiance. Since super-radiance implies the simultaneous decay of the majority of qubits, one can consider this super-radiance as a cause of error. Lemberger and Yavus analyzed how the decay rate of only certain qubits is affected by other qubits based on the above theory. In order to make the features easier to see, the initial state is set as follows.

(21) $$|\psi \left(t=0\right)\rangle =\sum_{m=0}^{2^{N-1}-1}c_{m,0}{|g\rangle }_{j}⊗|m,0\rangle +\sum_{m=0}^{2^{N-1}-1}d_{m,0}{|e\rangle }_{j}⊗|m,0\rangle$$

where *m* corresponds to an indicater of the quantum state of a qubit of N−1 other than *j*th qubit. If the density operator on the composite space is ρ=|ψ〉〈ψ| , then the density operator of *j*th qubit is obtained by tracing this density operator over a qubit fraction of N−1 . The result is

(22) $$\begin{array}{ccc} \rho_{j} & = & \sum_{m=0}^{2^{N-1}-1}|c_{m,0}{|}^{2}{|g\rangle }_{jj}\langle g|+\sum_{m=0}^{2^{N-1}-1}|d_{m,0}{|}^{2}{|e\rangle }_{jj}\langle e|\\ & & +\sum_{m=0}^{2^{N-1}-1}c_{m,0}d_{m,0}^{*}{|g\rangle }_{jj}\langle e|+\sum_{m=0}^{2^{N-1}-1}c_{m,0}^{*}d_{m,0}{|e\rangle }_{jj}\langle g| \end{array}$$

In the initial state, if the qubit of *j* is excited, *N* qubits radiate at once. On the other hand, if it is on the ground, the qubits of N−1 radiate at the same time. As a result,

(23) $$\begin{array}{ccc} & & |c_{m,0}{\left(t\right)|}^{2}∼{\left|c_{m,0}\left(t=0\right)\right|}^{2}e^{-({\left(N-1\right)}^{2}/4)\Gamma t} \end{array}$$

(24) $$\begin{array}{ccc} & & |d_{m,0}{\left(t\right)|}^{2}∼{\left|d_{m,0}\left(t=0\right)\right|}^{2}e^{-(N^{2}/4)\Gamma t} \end{array}$$

where Γ is a function of $\eta^{*}$ . From the above, Lemberger and Yavus [11,12] showed that as the subsection IV *A*, the error probability of the *j*th qubit at gate time δt can be described by

(25) $$p\left(error\right)=f\left(\eta^{*},N\right)∼\frac{1}{2}\Gamma N^{2}<\frac{1}{2}$$

The above formula is valid when Γ≪1 , because of physical approximation. This phenomenon is non-local. I would like to emphasize that this causes a burst error to the whole system with the same probability as the above equation.

### 4.3. Leak from Decoherence Free Subspace Due to Collective Decoherence

One can use a decoherence free subspace (DFS) [22] to avoid an error in the system that interacts with a heat bath. The evolution for the traced density operator is given by the Lindblad equation in general. Let the Hilbert space of the system of quantum bits be $H_{S}$ and all density operators on it be $D(H_{S})$ .

**Definition** **1.** *A decoherence free subspace : $H_{DFS}$ is a subspace of $H_{S}$ in which all density operators $\rho \in D(H_{DFS})$ defined in that space satisfy the following equation.*

(26) $$\frac{\partial \rho }{\partial t}=\frac{-i}{\hbar }\left[H,\rho \right]\;\forall t$$

In other words, it is equivalent to the absence of the effect of the Lindblad operator in Equation (8). The alternative way of characterizing the DFS is to consider all possible of singlet states

(27) $${|\Psi \left(j,k\right)\rangle }_{DFS}=\frac{1}{\sqrt{2}}{(|e\rangle }_{j}⊗{|g\rangle }_{k}-{|g\rangle }_{j}⊗{|e\rangle }_{k}$$

However, Lemberger and Yavus pointed out that there exists a phenomenon of the leak from DFS when collective decoherence occurs [11,12]. The decay rate of the stochastic amplitude at this time is interpreted as the rate at which the system leaks from the decoherence free subspace into a large extended Hilbert space. As a result, the leak probability is regarded as follows.

(28) $$P_{\left(Leak\right)}\left(t\right)∼\Gamma \delta tN^{2}$$

where Γ≪1 . This causes also nonlinear error depending on the number of qubits, and also it gives the burst error.

## 5. Communication Channel Modeling of Quantum Errors Due to Quantum Correlation

In the former sections, I have introduced new error phenomena due to quantum noise which depend on the number of qubits. Although the main concern in physical analysis is to discuss the decay rate by interaction with environments, in the discussion of the information theory for error correction, one needs to know the feature of error probability of information by the decay process. Phenomena of quantum decay by quantum noise depending on the number of qubits means the increasing of error probability by a quantum correlation. There is no such phenomenon in the conventional information theory. In this section, I give a model for error phenomena due to the above quantum phenomena by using classical probability. As a result, researchers of information theory can well understand such strange error phenomena in quantum computers.

### 5.1. Semi-Classical Modeling of Quantum Bit Array Structure

When a group of qubits is placed in a given environment, it was pointed out in the above section that an increase in the number of qubits enhances the error probability of one of its components. Here, I attempt to describe such quantum phenomena using only information theoretic concepts [15,16,17], leaving out the physical processes. Since our concern is to characterize the error probability, the causes of error such as bit error, phase error, entanglement error, and so on, are not considered. In this case, one can think of a qubit as just a bit, and a model as a two-dimensional arrangement of bits and the interaction of error factors from the environment with the qubits.

Let us assume that i,j are positions of *N* qubits on the two dimensional surface. Then, let us describe the qubits by the information bit $x_{\left(m,n\right)}$ of the spatial position (m,n) . And $e_{\left(m,n\right)}$ means the error bit for that information bit. So one can employ the following representation:

(29) $$\left(\begin{array}{c} x_{\left(1,1\right)}⨁e_{\left(1,1\right)},\ldots x_{\left(1,N\right)}⨁e_{\left(1,N\right)}\\ x_{\left(2,1\right)}⨁e_{\left(2,1\right)},\ldots x_{\left(2,N\right)}⨁e_{\left(2,N\right)}\\ \vdots \ldots \\ x_{\left(N,1\right)}⨁e_{\left(N,1\right)},\ldots x_{\left(N,N\right)}⨁e_{\left(N,N\right)} \end{array}\right)$$

The quantum correlations among qubits are described by the coupling probability $p_{\left(m^{*},n^{*}\right),\left(m,n\right)}\equiv p_{j,k}$ , j,k∈N among error bits in this modeling. Here I emphasize the fact that only the probabilistic nature of the error is an essential factor as the first stage in the information theoretic analysis. Of course, in the design stage of the quantum error correction scheme, one needs more detailed physical characterization. However, this is beyond the scope of this paper. The reason is that if the kind of noise discussed here occurs, consideration of the error correction mechanism loses its meaning.

### 5.2. Semi-Classical Description of Nonlinear Local Correlated Errors

I discuss here the nonlinear errors due to local quantum correlation. One qubit at center position $x_{\left(m^{*},n^{*}\right)}$ in two dimensional space is prepared and several qubits $N_{sub}$ of *N* are put around the first qubit with quantum mechanical correlation. Let us assume that the decay rate of the center qubit is assumed as $\eta^{*}$ when it is a single. If the error probability of the center qubit due to the interaction among other quabits is given by

(30) $$p\left(error\right)=f\left(\eta^{*},N_{sub}\right),$$

then this is the nonlinear error due to local quantum correlation. To verify this feature of error, I deal with the recurrence effect introduced in the section IV *A* . Let us assume that the Hutter–Loss effect occures in the quantum processor. Hutter–Loss [13], as the first step of the recurrence effect, gave a semi-classical description of error performance for own phenomena on the recurrence effect as follows. The probability of error of the center qubit is $\eta_{m,n}=\eta^{*}$ . Let $N_{sub1}=5$ be a subset of qubits which are set around the first qubit . Let the latent probability (correlation) of an error-induced in pairwise with the center and one of four qubits be $0\leq p_{\left(m^{*},n^{*}\right),\left(m,n\right)}\equiv p_{1}^{*}\leq 1/2$ . In this case, the error probability of the center qubit in subset ( $N_{sub1}=5$ ) is given by the following [13]:

(31) $$\begin{array}{ccc} & & p\left(error\right)=\eta^{*}\sum_{q:even}\frac{4!}{q!(4-q)!}{\left(p_{1}^{*}\right)}^{q}{\left(1-p_{1}^{*}\right)}^{4-q}\\ & & +\left(1-\eta^{*}\right)\sum_{q:odd}\frac{4!}{q!(4-q)!}{\left(p_{1}^{*}\right)}^{q}{\left(1-p_{1}^{*}\right)}^{4-q}\\ & & =\frac{1}{2}-\frac{1}{2}\left(1-2\eta^{*}\right){\left(1-2p_{1}^{*}\right)}^{4}=\frac{1}{2}-\frac{1}{2}\left(1-2\eta^{*}\right)\Lambda_{1}\\ & & \equiv \eta_{1}^{*}\geq \eta^{*} \end{array}$$

where $\Lambda_{1}={\left(1-2p_{1}^{*}\right)}^{4}$ . This is an example of the nonlinear error, because it is greater than $\eta^{*}$ . This shows that the error is not changed when $p_{1}^{*}=0$ . It means that there is no correlation among qubits.

The above is just one step for the scalable system. To understand the recurrence phenomena, let us consider a more complicated structure of correlated qubits in general processors, with an extension of the above formula. Let us add four qubits to the initial five qubits, and let the latent probability (correlation) of an error-induced in pairwise with the center and one of new four qubits be $0\leq p_{2}^{*}\leq 1/2$ , respectively, based on operation gate such as control NOT. From the recurrence phenomena, the error probability for the qubit in the center has to employ the initial probability $\eta_{1}^{*}$ given by the Equation (31) instead of $\eta^{*}$ . That is, $\eta^{*}$ is replaced by $\eta_{1}^{*}$ , and $p_{1}^{*}$ is replaced by $p_{2}^{*}$ in Equation (31). Then one gets the following

(32) $$\begin{array}{ccc} & & p\left(error\right)=f\left(\eta_{1}^{*},N_{sub2}\right)=\eta_{1}^{*}\sum_{q:even}\frac{4!}{q!(4-q)!}{\left(p_{2}^{*}\right)}^{q}{\left(1-p_{2}^{*}\right)}^{4-q}\\ & & +\left(1-\eta_{1}^{*}\right)\sum_{q:odd}\frac{4!}{q!(4-q)!}{\left(p_{2}^{*}\right)}^{q}{\left(1-p_{2}^{*}\right)}^{4-q}=\frac{1}{2}-\frac{1}{2}\left(1-2\eta_{1}^{*}\right){\left(1-2p_{2}^{*}\right)}^{4} \end{array}$$

Here $\Lambda_{2}={\left(1-2p_{2}^{*}\right)}^{4}$ . So, the above becomes as follows:

(33) $$p\left(error\right)=f\left(\eta^{*},N_{sub2}\right)=\frac{1}{2}-\frac{1}{2}\left(1-2\eta^{*}\right)\Lambda_{1}\Lambda_{2}$$

Let us repeat the same operation. One needs to replace the initial probability and the latent probability of an error-induced in pairwise in each operation. Finally one gets the following

(34) $$p\left(error\right)=f\left(\eta^{*},N_{subK}\right)=\frac{1}{2}-\frac{1}{2}\left(1-2\eta^{*}\right)\prod_{l=1}^{K}\Lambda_{l}$$

where $\Lambda_{l}={\left(1-2p_{l}^{*}\right)}^{4}$ , and l={1,2,⋯,K} . When one constructs the structure based on the steps of *K* times, the error probability of each qubit increases with respect to *K*. This model provides a visualization of the new type of quantum error due to the recurrence effect, and clarifies a curious feature of the nonlinear error. That is, despite the nature of the quantum noise from the environment as being invariant, the probability of its own error increases when qubits are clustered together. As a special case, when *K* is increased, one has following characteristics:

(35) $$\begin{array}{ccc} p(error) & = & f\left(\eta^{*},N_{subK}\right)=\eta^{*},\;p_{l}^{*}=0\;\forall l \end{array}$$

(36) $$\begin{array}{ccc} p(error) & = & \eta_{j}\left(N_{subK}\right)\to \frac{1}{2},\;p_{l}^{*}\neq 0,K\gg 1 \end{array}$$

### 5.3. Semi-Classical Description of Nonlinear Non-Local Correlated Errors

The decoherence by nonlocal correlation among all qubits such as super radiance is alsoa serious phenomena in quantum processors. This phenomenon was explained in Section 4.2. The problem is how to describe such a decoherence based on a communication channel model for information theory. It is known that a qubit can be classically viewed as a radiating dipole at the frequency of the qubit transition. This radiating dipole produces an electric field at the position of the qubit. This electric field interacts with whole qubits and induces unwanted rotation of the qubit state. The decay rate of dephasing by this rotation can be modeled by the Rabi frequency due to the electric field. The value is proportional to the electric field. The Rabi frequency of the *j*-th qubit affected by all qubits is given by the incoherent sum from each emission of the other qubits.

Here I give a model for nonlinear error and burst error in the case when a qubit at a certain position $x_{\left(m^{*},n^{*}\right)}$ of Equation (29) interacts, based on the quantum mechanical method with whole qubits. Let the qubit at $x_{\left(m^{*},n^{*}\right)}$ be the *j*th qubit. Then, $\gamma_{m^{*},n^{*}}\equiv \gamma_{j}^{*}$ is the decay rate of the qubit at $x_{m^{*},n^{*}}$ due to an effect of that interaction, and let us assume that $N_{super}$ is the number of qubits in a region of super radiance. Then, the interaction efficiency amongst all the qubits can be described by $0\leq \nu_{\left(m^{*},n^{*}\right),\left(m,n\right)}\equiv \nu_{j,k}\leq 1$ , where $j,k\in N_{super}$ .

The decay rate of the *j*th qubit due to the interaction between the position $x_{m^{*},n^{*}}$ and all the existing qubits is modeled as the sum of each decay rate from an analogy of Rabi oscillation coupling as follows:

(37) $$\gamma_{j}\left(N_{super}\right)=\sum_{k=1,k\neq j}^{N_{super}}\nu_{j,k}\gamma_{j}^{*}$$

Thus, the error probability of *j*th qubit due to its interaction during Δt is defined as follows:

(38) $$p\left(error\right)=\frac{1}{2}\left(1-\exp\{-\right|\gamma_{j}\left(N_{super}\right){|}^{2}\})\;\forall j$$

As one can see, the above model provides a way to understand the origin of nonlinear effects in the error performance, which depends on the number of qubits. When $\nu_{j,k}=1,\forall j,k$ , it corresponds to the Lembeger–Yavus super radiance decay, which is introduced in Section 4.2. Let us consider the physical counterpart in Section 4.2 of the above equation. The correspondence between $\gamma_{j}^{*}$ and the physical decay rate Γ corresponds to ${\left(\gamma_{j}^{*}\right)}^{2}=\Gamma$ . When Γ≪1 (good quality), and there is no super radiance, the error probability is nearly zero. However, even if ${\left(\gamma_{j}^{*}\right)}^{2}=\Gamma \ll 1$ , when the super radiance occurs, the concrete form of the Equation (42) can be approximated as follows:

(39) $$p\left(error\right)≅\frac{1}{2}\Gamma N_{super}^{2}<\frac{1}{2}$$

It is valid for Γ≪1 and for finite $N_{super}$ . This matches the result of the Lembeger–Yavus super radiance decay. On the other hand, when the number of qubits of super radiance is large, or Γ is large, the error probability goes to 1/2.

In addition, when super radiance occurs, the correlation consists of all the qubits. This fact drives a serious error such as burst error, in which all qubits are destroyed simultaneously with the following probability:

(40) $$P_{e}\left(burst\right)≅\frac{1}{2}\Gamma N_{super}^{2}$$

## 6. Communication Channel Modeling of Quantum Error Due to External Forces

### 6.1. Physical Reality of External Force Such as Cosmic Rays

I have been discussing the decay effects of the interaction of a particular qubit with the environment in a quantum processor. On the other hand, a similar error occurs through interaction with external forces by particles coming from outside the systems. These include cosmic ray from space, γ -ray and charged particles from laboratory environments. Occurrence of such a case was pointed out by Vepsalainen et al. [23] in the case of superconducting quantum computers. Here let $p_{ex}$ be the probability that one qubit in the system is hit by an external force. In this case, the error probability in our model Equation (1) for one qubit is modified by replacing the initial error rate $\eta^{*}$ as follows:

(41) $$\eta_{ex}^{*}=\eta^{*}+p_{ex}$$

When this occurs, a burst error occurs in addition to the above. This phenomenon has been experimentally demonstrated in superconducting quantum computers by Wilen et al. [24]. In this section, let us discuss a model for a burst error by such phenomena.

### 6.2. Communication Channel Error Model Due to Environment Correlation

In Section 4, I discussed the correlated error by quantum correlation and clarified the formulation of nonlinear error. There are no such phenomena in the classical world. Even if there is no quantum correlation among the qubits, the correlation among the qubits exists in superconducting quantum computers. That is, the charge field of qubits or other parameters may have the potential to generate a correlation, or the environment itself may generate the correlation among qubits. In fact, the discovery of long-range two-qubit correlations has been reported [24,25]. Thus, one has to consider the communication channel model for error propagation of qubits due to classical correlation. Although the physical phenomena of the interaction between qubits and environment are very complicated, let us simplify this situation. Assume that $p_{\left(k|j\right)}$ is the conditional probability that qubit *k* is affected when an error of qubit *j* is caused by an external force. The total scheme is described on two dimensional constellation of qubits by

(42) $$\left(\begin{array}{c} p_{\left(1|1\right)},\ldots ,p_{\left(1|N\right)}\\ p_{\left(2|1\right)},\ldots ,p_{\left(2|N\right)}\\ \vdots \ldots \\ p_{\left(N|1\right)},\ldots ,p_{\left(N|N\right)} \end{array}\right)$$

These conditional probabilities correspond to correlations among electric charge fields of qubits.

Here the burst error caused by external force is described as follows: One of the qubits makes an error due to a collision with an external particle, then the other many qubits make errors in conjunction with that error. Let us deal here with a simple example. If the ripple effect on qubits is the most simple, then the burst probability is given by only conditional probabilities on the *j*-th qubit as follows:

(43) $$P\left(burst\right)=\eta_{ex}^{*}\prod_{k\neq j}p\left(k|j\right)\;\forall k\;p\left(k|j\right)\neq 0$$

The other is a chain of errors like a Markov chain, in which the error in the *j*-th qubit propagates to the *i*-th qubit, and then the error in the *i*-th qubit propagates to the *k*-th qubit, and so on. One can define it as the avalanche burst effect. In this case, the avalanche burst probability can be approximated by

(44) $$P\left(burst\right)∼\eta_{ex}^{*}p^{*}\left(k|j\right)p^{*}\left(l|k\right)\cdots$$

where $p^{*}\left(k|j\right)$ implies the maximum probability of the transition from *j* to *k*. The parameters to control such effects are $\eta^{*}$ , and the correlation through the electric field around the superconducting qubit. If the ripple effect is more complex, then one should consider all the qubits in quantum processors to be collapsing. However, the experimental results of error propagation in the reference [23,24,25] may be analyzed with the above modeling.

In order to reduce such effects, one should design the circuit such that correlations among electric charge fields of qubits in the superconducting quantum computers are eliminated.

## 7. Communication Channel Modeling of Quantum Error in Operations

If an initial quantum state of a qubit decays to the vacuum state, it becomes an error of the quantum computer. So one can consider the physical protection of the decay of qubits to avoid such errors. The quantum Zeno effect is a typical example of a methodology for keeping a qubits in a normal state [26,27]. The use of this phenomena to stabilize qubits in quantum processors has been proposed by Franson’s group [28]. In this section, we analyze the external noise effect for such artificial operations.

### 7.1. Collapse of Quantum Zeno Effect for Single Qubit

In conventional theory with the ideal environment, the quantum Zeno effect is described by the survival probability of the initial state as follows [26,27]:

(45) $$P\left(\delta t\right)=\left|\langle \psi \right|e^{-iHt}{\left|\psi \rangle \right|}^{2}\approx 1-{\left(\frac{\delta t}{\tau_{z}}\right)}^{2}$$

where δt≪1 , *H* is the total Hamiltoniann of the system and the Zeno time is $\tau_{z}^{2}=\langle \psi |H_{int}^{2}|\psi \rangle$ . Then the survival probability at time *t* after the *J*th measurements is

(46) $$P_{\left(J\right)}\left(t\right)=P{\left(\delta t\right)}^{J}\approx {\left[1-{\left(\frac{\delta t}{\tau_{z}}\right)}^{2}\right]}^{J}$$

If one assumes that *t* is fixed and the time interval δt=t/J , one has a convenient formula of the above relation as follows:

(47) $$P_{\left(J\right)}\left(t\right)=P{\left(\delta t\right)}^{J}\approx 1-\frac{1}{J}\left(\frac{t^{2}}{{\tau_{z}}^{2}}\right)\to 1,\;1\ll J$$

Thus the initial state is kept. This is called the quantum Zeno effect. However, in the general or non ideal environment of quantum computers, it has been pointed out that the quantum Zeno effect may be eliminated.

Since my purpose is to formulate in the sense of information theory, I do not describe the exact physical model. I employ here the stochastic Schr $\ddot{o}$ dinger equation discussed by Adler and Diosi ([29], also see Appendix B).

(48) $$d|\Psi \rangle =-iH|\Psi \rangle dt-V_{r}^{2}{\left(H-<H>\right)}^{2}|\Psi \rangle dt+V_{r}\left(H-<H>\right)|\Psi \rangle dW_{t}$$

where *H* is the Hamiltonian, <H> =〈Ψ|H|Ψ〉 . $V_{r}$ is a parameter governing the strength of an external force. Here one can employ the standard theory of the stochastic processes [30,31]. $dW_{t}$ is an It $\hat{o}$ stochastic differential together with dt , and it obeys the Standard It $\hat{o}$ calculus rules as follows:

(49) $$dW_{t}^{2}=dt,\;dW_{t}dt=dt^{2}=0$$

where the Wiener process is

(50) $$W_{t}=\int_{0}^{t}dW_{t}$$

In It $\hat{o}$ calculus, the following formula is used.

(51) d(AB)=(A+dA)(B+dB)−AB=(dA)B+AdB+dAdB

Thus, the next relation is given.

(52) $$d\exp\left(bW_{t}\right)=\exp\left(bW_{t}\right)<bdW_{t}+\frac{1}{2}b^{2}dt>$$

where *b* is a real number. Then one has

(53) $$\begin{array}{ccc} & & d<\exp\left(bW_{t}\right)>\;=\;<\exp\left(bW_{t}\right)>\frac{1}{2}b^{2}dt \end{array}$$

(54) $$\begin{array}{ccc} & & <\exp\left(bW_{t}\right)>\;=\exp\left(\frac{1}{2}b^{2}t\right) \end{array}$$

The equation for the density operator ρ=|ψ〉〈ψ| is given by

(55) $$\begin{array}{ccc} & & d\rho =(d|\psi \rangle )\langle \psi |+|\psi \rangle d\langle \psi |+d|\psi \rangle d\langle \psi |\\ & & =i\left[\rho ,H\right]dt-V_{r}^{2}\left[H,\left[H,\rho \right]\right]dt+V_{r}\left[\rho ,\left[\rho ,H\right]\right]dW_{t} \end{array}$$

Based on the above equation, the Adler and Diosi gave the following formula [29].

(56) $$P\left(\delta t\right)\approx 1-\frac{1}{\tau_{z}^{2}}\left(V_{r}^{2}\delta t+{\delta t}^{2}\right)$$

Let us assume here that *t* is fixed. One can describe the survival probability at the artificial operations as follows:

(57) $$P_{\left(J\right)}\left(t\right)=P{\left(\delta t\right)}^{J}\approx 1-\left\{\frac{{V_{r}}^{2}}{{\tau_{z}}^{2}}t+\frac{1}{J}\left(\frac{t^{2}}{{\tau_{z}}^{2}}\right)\right\}$$

The survival probability decreases with respect to *t* even if one operates any measurement, because the second term is independent of *J*. Following the above results, one can formulate the system failure probability of the artificial operation to protect the qubits as follows:

(58) $$P_{f}=1-P{\left(\delta t\right)}^{J}\approx \frac{{V_{r}}^{2}}{{\tau_{z}}^{2}}t\;<1$$

The above formulae are valid only for t≪1 in the perturbation approximation in physical analysis. As a result, the failure performance is mainly described by a single parameter $V_{r}$ . This is very useful for information theorists who are not interested in the detailed physical process.

### 7.2. Collaps of Quantum Zeno Effect for Qubits with Correlation

Here I discuss the scheme of a system of several qubits with quantum correlation, such as entanglement. In general, when the conventional environment is employed, an entangled state between qubits j=1 and 2 in memory can be protected by applying the Zeno effect in a composite system of qubits and environment. That is, let us consider a series of non selective measurements on the qubits performed at time interval δt . These have the following properties. One is the projection onto the collective ground state $|\phi {>}_{G}=\left|0{>}_{1}\right|0{>}_{2}$ , and the other is that the measurement cannot distinguish between $|1{>}_{1}|0{>}_{2}$ and $|0{>}_{1}|1{>}_{2}$ . These measurements disentangle the qubits from the environment at each time δt . The survival probability is given by the same formula Equation (61) of the case of single qubit.

Let us consider the non-ideal environment. I consider the *N* qubits system with quantum correlation with each other. When one employs a general Zeno effect operation on all the qubits, if the environment for all qubits is the ideal case, one can use the protection scheme of the system. Let us assume that an environment becomes a general or non-ideal environment for a single qubit in *N* qubits. That is, the stochastic coupling parameter by external force $V_{r}$ for a certain qubit is non zero. Then, the qubit in the non-ideal environment suffers the same effect as the case of the single qubit, such as in Equation (58). Hence one cannot deny the possibility that the whole system is destroyed, because all the qubits have quantum correlations. Thus, the system failure probability for the whole system can be evaluated by

(59) $$P_{f}\left(Nqubits\right)\approx \frac{{V_{r}^{*}}^{2}}{{\tau_{z}}^{2}}t\;<1$$

This is valid for t≪1 . The concrete value of $V_{r}^{*}$ should be determined by the concrete physical analysis, but the physical structure is not so important for the information theory.

## 8. Conclusions

In this paper, a new type of error performance, the so called nonlinear error, where the error probability for a single qubit depends on the number of qubits in the system, has been discussed. I have shown how to model such strange properties of error probability based on a semi-classical method. Then it has been clarified that nonlinear errors give serious degradations of the capability of quantum computer, by the recurrence effect due to quantum correlation and also by collective decoherence . In order to cope with the quantum errors described in this paper, or to avoid this situation, one method is to further develop the conventional quantum error correction theory based on quantum noise analysis, or to establish a new way to physically suppress such errors [32,33,34]. Recently, a number of previously unknown and extremely difficult challenges in the development of an error correctable quantum computer have been reported [35,36,37,38]. However, I believe that the ideal quantum computer will be realized in the future. I expect that this paper may provide some hints for finding a way toward the ideal quantum computer.

Finally, I would like to point out that it is difficult to predict the realization of a quantum computer capable of cryptanalysis. However, because my results suggest that the capability of a real quantum computer is strictly limited, one can say that the current cryptography is not subject to the danger posed by current quantum computers. However, one should develop quantum computer-resistant cryptosystems based on mathematical analysis [39,40], or by physical cipher on the assumption that an ideal quantum computer or new mathematical discovery can be realized in the future.

## Appendix A. Physical Research of Decoherence and Disentanglement Phenomena

The decoherence issue is of great significance for the foundations of quantum physics, as well as for problems of practical interest, such as quantum engineering. In the past two decades, it has become increasingly clear that many of the symptoms of classicality can be induced in quantum systems by their environments. Furthermore, issues of disentanglement and quantum discord are the same subjects as decoherence. The fundamental discussions on these physical phenomena have been given by Zurek [41] and Yu et al. [42,43]. The mathematical foundation for such issues belongs to the open system theory in quantum mechanics. The detailed analysis based on the open system theory for recent topics have been given by Lo Franco et al. [44], de Vega et al. [45], Bellomo et al. [46], and Aaronson et al. [47], respectively. Moreover, the experimental justification has been discussed by Rotter et al. [48]. Readers who are interested in the physical problem of decoherence and quantum noise in quantum processing, including quantum computing, can obtain detailed scientific knowledge from the above references.

## Appendix B. From Lindblad Equation to Semi-Classical Stochastic Differential Equation

In general, dissipative processes in physical systems are governed by complete positive maps, and dynamical semigroup theory plays an important role. Complete positive maps $\hat{X}\to T\left(\hat{X}\right)$ is described by a Kraus representation $T\left(\hat{X}\right)=\sum A_{n}\hat{X}A_{n}$ . However, in order to analyze dynamic processes, it is necessary to shift the mathematical system to infinitesimal analysis. The time evolution of a fully positive statistical operator is described by the following Lindblad equation, which is an embodiment of Equation (8).

(A1)
$$L\rho =\frac{-i}{\hbar }\left[H,\rho \right]+\sum_{n\in N}\left[v_{n}\rho v_{n}^{†}-\frac{1}{2}\left\{v_{n}^{†}v_{n},\rho \right\}\right]$$

The relationship between the $v_{n}\rho v_{n}^{†}$ terms can be understood by identifying $v_{n}$ : the fast-varying component of $A_{n}$ with a strength of ${\left(dt\right)}^{1/2}$ . This fact may enable us to understand the Lindblad equation in the context of stochastic differential equations [29]. From the standard treatment of quantum noise [31], one can begin by mapping $A_{n}$ to the following

(A2)
$$\begin{array}{ccc} A_{n} & = & d_{n}+u_{n}dt+v_{n}dW_{t}^{n}\\ A_{n}^{†} & = & d_{n}+u_{n}^{†}dt+v_{n}^{†}dW_{t}^{n} \end{array}$$

where $d_{n}$ is a positive number and, *W* is the Wiener process. $dW_{t}^{n}$ has the following properties from It $\hat{o}$ calculus.

(A3)
$$dW_{t}^{m}dW_{t}^{n}=c^{mn}dt,\;dW_{t}^{m}dt=0$$

where *c* is real symmetric covariant matrix, and its diagonal elements are $e^{nm}=1,\forall n$ . Let us consider that the complete positive map is described by

(A4)
ρ→ρ+dρ=T(ρ)

The we have the following relation from the above explanations.

(A5)
$$\begin{array}{ccc} & & \rho +d\rho =\sum_{n\in N}\left(d_{n}+u_{n}dt+v_{n}dW_{t}^{n}\right)\rho \left(d_{n}+u_{n}^{†}dt+v_{n}^{†}dW_{t}^{n}\right)\\ & & =\sum_{n\in N}d_{n}^{2}\rho +\sum_{n\in N}d_{n}\left(v_{n}\rho +\rho v_{n}^{†}\right)dW_{t}^{n}+\left(\rho U^{†}+U\rho +\sum_{n\in N}v_{n}\rho v_{n}^{†}\right)dt \end{array}$$

where $U=\sum_{n\in N}d_{n}u_{n}$ , $\sum d_{n}^{2}=1$ . Then we have the form of the stochastic differential equation as follows:

(A6)
$$d\rho =\sum_{n\in N}d_{n}\left(v_{n}\rho +\rho v_{n}^{†}\right)dW_{t}^{n}+\left(\rho U^{†}+U\rho +\sum_{n\in N}v_{n}\rho v_{n}^{†}\right)dt$$

When one takes the partial trace with respect to the disturbance, it becomes as follows:

(A7)
$$dE\left[\rho \right]=E\left[d\rho \right]=(E\left[\rho \right]U^{†}+UE\left[\rho \right]+\sum_{n\in N}v_{n}E\left[\rho \right]v_{n}^{†})dt$$

This is equivalence to the Lindblad equation as follows:

(A8)
$$\frac{dE[\rho ]}{dt}=\frac{-i}{\hbar }\left[H,E\left[\rho \right]\right]+\sum_{n\in N}\left(v_{n}E\left[\rho \right]v_{n}^{†}-\frac{1}{2}v_{n}^{†}v_{n}E\left[\rho \right]-\frac{1}{2}E\left[\rho \right]v_{n}^{†}v_{n}\right)$$

where $v_{n}$ , $v_{n}^{†}$ correspond to Lindblad operator.
